# Brachial plexus avulsion neuropathic refractory pain: association of spinal cord stimulation and DREZotomy for complex pain

**DOI:** 10.3171/2020.7.FOCVID2035

**Published:** 2020-10-01

**Authors:** Rômulo A. S. Marques, Rodrigo A. C. Cavalcante, Lucas M. C. E. Pimenta

**Affiliations:** 1 Department of Neurosurgery and; 2 School of Medicine, Federal University of Goiás, Goiânia, Goiás, Brazil

**Keywords:** neuropathic pain, brachial plexus avulsion, dorsal root entry zone

## Abstract

A 52-year-old male was involved in a car accident 30 years ago. He presented with complete motor paralysis of the left upper limb. This evolved into severe neuropathic pain (mainly shocking and burning sensation) distributed from C5 to T1 dermatomes. He was first treated with spinal cord stimulation (SCS), which did not show efficacy for pain control, maintaining high visual analog scale (VAS) scores. He then received complementary treatment with left cervical DREZotomy to mitigate suffering and preserve SCS function to control “mirror pain.”

The video can be found here: https://youtu.be/iTvbLAZ3odM

## Transcript

**0:26 Clinical Presentation: Part 1.** This is a 52-year-old male patient who had a car accident 30 years ago. On physical examination, he presented a complete motor paralysis of the left upper limb with a severe muscle atrophy, as you can see in the picture. He evolved into a severe neuropathic pain, mainly shocking and burning sensation, distributed from C5 to T1 dermatomes.

**0:50 Clinical Presentation: Part 2.** The electroneuromyography revealed a complete lesion at the C5 and C6 levels and partial lesion at the C7, C8, and T1 levels. These findings were compatible with a brachial plexus avulsion. For years, the patient has been suffering a complex neuropathic pain, which has not shown adequate control with medicines and adjuvant therapies (visual analog scale 7–9). A spinal cord stimulation placed at the C3–4 levels was carried out 3 years ago in order to relieve the pain, but it presented a poor pain control using tonic and then high-density stimulation. There was mild reduction to VAS 5–8.

**1:37 Surgical Strategy.** The pain control was unsatisfactory, causing him a striking discomfort and emotional suffering. The inclusion criteria for DREZotomy was neuropathic pain, VAS greater than 5, sleeping problems, high doses of strong opioids such as methadone, and anticonvulsant such as gabapentin for more than 1 year.^1–3^ He failed pain control doing autonomic blockage and neurostimulation. After the failed attempts to control pain, a dorsal root entry zone [DREZ] ablation was pointed out.^1,4,5^

**2:10 Surgical Strategy: Form of Lesion.** The microsurgical approach has low rate of corticospinal and dorsal column deficit, with efficacy of 87% of good results.^6–8^ Radiofrequency thermocoagulation lesion report complications between 0% and 60%, and a mean pain relief of 85% to 80% of the patients.^1,6,9^ Both techniques seem to be even in good results and complication rate, so we chose our service routine. No evidence of infection or coagulation dysfunction were found.

**2:44 Patient Positioning.** Patient is positioned in prone with the head placed in the Mayfield head holder with a mild flexion.

**2:50 Skin Incision.** We make an enlargement of the previous incision’s opening on a midline posterior approach from C2 to T2 in order to get through the spinal cord electrode placement and T1 medullar segment.

**3:04 Protection of the Electrode.** To protect the electrode, we slide it up from C3–4 to C2 (the electrode is positioned from the top down on the epidural plane).

**3:18 Laminotomy.** The laminotomy is completed using a high-speed drill—hence a minimal bone loss and wide opening.

**3:24 Neurosurgical Opening Technique.** Hemostatic agents are placed at the epidural space to obviate further epidural bleeding. The dura mater is opened in standard fashion under microscope. Dural sutures are placed for better exposure.

**3:37 Dissection to Identify Existing Pathology.** The arachnoid membrane is separately dissected using microscissors. It seems thicker than usual, suggesting a previous traumatic spinal cord and fibrosis injury. Spinal cord distortion and neovascularization were found. Before thermocoagulation we performed Nashold’s technique to identify the posterior lateral sulcus:^1,9,10^ 1) Noted upon closer inspection, the avulsion of the C5, C6, and C7 dorsal rootlets, exposing naked dorsal root entry zones on the left side. A tortuous pial vein runs on the posterolateral sulcus. 2) Inspection of remaining dorsal roots of C4 and C8. 3) Palpation of the spinal cord with a fine needle (lateral sulcus is easily indented).^1,9^

**4:38 Treatment of the Underlying Pathological Process: Part 1.** The DREZ access was prepared with microsurgical dislocation of some parts of the posterior lateral sulcus vein, and some of the radicular adhesions.

**5:04 Treatment of the Underlying Pathological Process: Part 2.** We covered the left dorsal root entry zone from C5 to T1 with radiofrequency lesions point-to-point 2-mm distance between them, with 45° angled needle.^1,9,10^ We used a cordotomy needle 2-mm-depth electrode, and its temperature is elevated to 65°C through 30 seconds (or we stop lesioning when tissue retraction starts),^1,9,10^ aiming that these lesions of the dorsal horn coalesce.^1,9^ The mean number of lesions is about 45–50 at the end of the procedure. Eventual bleeding was treated with warm saline irrigation.

**5:56 Closure Techniques.** The dura is closed in a watertight fashion and sealed using biological glue and dural patch. The bone of laminoplasty is returned to its place and fixed with miniplates and 4-mm miniscrews in each lamina.

**6:10 Positioning Back the Electrode.** We slide back the electrode to the preop position and fix it again. The impedanciometry reading is completed; it shows no open circuitry.

**6:19 Postoperative Images.** A cervical tomography was performed after procedure showing well-positioned plates and vertebral laminae; electrode return to its prior position.

**6:30 Postoperative Course: Part 1.** In the immediate postoperative period, the VAS score was zero, associated with intense but pleasant left limb and upper thorax hypoesthesia. A mild hypoesthesia in the groin area and left inferior limb was noted, and improved progressively from proximal to distal, with the return of normal sexual activity in 3 weeks. A mild hemiparesis (muscle strength grade 3–4), due to venous congestion and spinal cord hyperemia, developed and was rapidly resolved in the next 3 weeks.

**7:02 Postoperative Course: Part 2.** In the first postoperative week, a mirror pain started. Peripheral nerve lesions can activate contralateral changes in normal neuronal function through the humoral mechanism with a number of breakdown products transported via blood or CSF. This can cause an aseptic inflammatory reaction of immunocompetent cells and reactive changes in affected axons and their perikarya, which can affect contralateral homonymous neurons through commissural interneurons.^3^ Microglia and astrocytes are activated following a peripheral nerve injury and increase in immunoreactivity, leading to dorsal horn neuron sensitization, and correlates with the development of allodynic behavior.^3^ In animal models, the mirror phenomena with contralateral paw hypersensitivity demonstrates rates in the order of 40%–50% in pain control with tonic SCS parameters.^4^ The tonic SCS reproduced the animal model with partial pain control.^4^ When we changed the parameters for high-density stimulation (1200 Hz) with large pulse width 400 μsec and 2-V amplitude covering with paresthesia the painful area, the result was complete pain relief.

## Author Contributions

Primary surgeon: Marques. Editing and drafting the video and abstract: all authors. Critically revising the work: all authors. Reviewed submitted version of the work: all authors. Approved the final version of the work on behalf of all authors: Marques. Supervision: Marques, Pimenta.
